# Augmented superior rectus muscle transposition in management of defective ocular abduction

**DOI:** 10.1186/s12886-020-01779-1

**Published:** 2021-01-20

**Authors:** Mohamed F. Farid, Ahmed E. M. Daifalla, Mohamed A. Awwad

**Affiliations:** 1 Ophthalmology Department, Benha University, Benha, Egypt; 2 King’s College Hospital, London, UK

**Keywords:** Superior rectus transposition, Dual augmented transposition, Sixth nerve palsy, Esotropic Duane retraction syndrome

## Abstract

**Background:**

Superior rectus muscle transposition (SRT) is one of the proposed transposition techniques in the management of defective ocular abduction secondary to chronic sixth nerve palsy and esotropic Duane retraction syndrome (Eso-DRS). The aim of the current study is to report the outcomes of augmented SRT in treatment of Eso-DRS and chronic sixth nerve palsy.

**Methods:**

a retrospective review of medical records of patients with Eso-DRS and complete chronic sixth nerve palsy who were treated by augmented full tendon SRT combined with medial rectus recession (MRc) when intraoperative forced duction test yielded a significant contracture. Effect on primary position esotropia (ET), abnormal head posture (AHP), limitation of ocular ductions as well as complications were reported and analyzed.

**Results:**

a total of 21 patients were identified: 10 patients with 6th nerve palsy and 11 patients with Eso-DRS. In both groups, SRT was combined with ipsilateral MRc in 18 cases. ET, AHP and limited abduction were improved by means of 33.8PD, 26.5°, and 2.6 units in 6th nerve palsy group and by 31.1PD, 28.6°, and 2 units in Eso-DRS group respectively. Surgical success which was defined as within 10 PD of horizontal orthotropia and within 4 PD of vertical orthotropia was achieved in 15 cases (71.4%). Significant induced hypertropia of more than 4 PD was reported in 3 patients (30%) and in 2 patients (18%) in both groups, respectively.

**Conclusion:**

augmented SRT with or without MRc is an effective tool for management of ET, AHP and limited abduction secondary to sixth nerve palsy and Eso-DRS. However, this form of augmented superior rectus muscle transposition could result in high rates of induced vertical deviation.

**Supplementary Information:**

The online version contains supplementary material available at 10.1186/s12886-020-01779-1.

## Background

Lateral transposition of vertical recti (VRT), either full or partial tendon, is a well-established surgical strategy to overcome esotropia (ET) and defective ocular abduction associated with esotropic Duane retraction syndrome (Eso-DRS) and sixth nerve palsy [1, 2]. In many instances, this transposition is combined with recession of ipsilateral medial rectus muscle (MRc) to control larger deviations especially in cases with MR contracture [3]. To reduce the possibility of anterior segment ischemia, superior rectus transposition (SRT), either isolated or combined with MRc, was proposed for treatment of sixth nerve palsy and Eso-DRS [4, 5].

Over years, various modalities have been proposed to augment the effect of the transposition procedure, for instance; posterior scleral fixation suture (Foster) [6], muscle to muscle union suture (Wright) [7] and segment resection of vertical recti [8]. Dual-type augmentation, in which muscle to muscle union is coupled with posterior scleral fixation, has been recently proposed to augment partial VRT in treatment of chronic sixth nerve palsy [9]. The aim of this study is to evaluate the results of dually augmented SRT with or without MRc for correction of ET, abnormal head posture (AHP) and limited abduction associated with Eso-DRS and sixth nerve palsy.

## Methods

After approval from institutional review board of Benha University hospital obtained, medical records of patients with chronic defective ocular abduction, precisely Eso-DRS and chronic sixth nerve palsy who underwent augmented SRT with or without ipsilateral MRc in the pediatric ophthalmology unit, Benha University Hospital, from 2013 to 2018 were retrospectively reviewed. Patients were excluded from data analysis if they had previous strabismus surgery or if their postoperative follow up was less than 3 months.

In addition to patients’ demographics, data collection included preoperative and postoperative angle of ocular deviation, head turn and limitation of ocular duction. Intra and postoperative complications were also recorded including development of new deviations, either vertical or horizontal and the need for reoperations. Ocular alignment in the forced primary position was measured at distant and near fixation using prism and alternate cover testing but data analysis was performed using orthoptic assessment of distant deviation only. Head turn was assessed and documented using orthoptic goniometer with 5-degree scale while patient fixated a 6-m distant target. Limitation of ocular duction was assessed using a 6-point scale with − 6 refers to inability of the eye to move from the adducted position, − 5 refers to movement of the eye towards the midline but without being able to reach it, while − 4 refers to limited duction in which the eye reaches the midline but without being able to continue past it [10].

Cases of sixth nerve palsy were included only if they were more than 6 months duration and if the palsy was complete as evident by severe limitation of abduction (≥ − 4), preoperative active forced generation testing and floating saccades. In cooperative patients, fusion was assessed using Worth’s four-dot test and stereopsis was measured using Titmus fly test.

All surgeries were performed under general anesthesia by the same surgeon (MFF). Ipsilateral MR muscle was recessed via limbal conjunctival incision, in case of positive forced duction test, by an amount sufficient to relieve the restriction, which usually did not exceed 5.5 mm, to avoid postoperative induced limitation of adduction. Surgical procedure of dual augmentation of SR muscle was performed following the steps previously described by Farid [9]. The SR muscle was reached via limbal incision and was then cleared from intermuscular septa and Tennon’s. Care was taken to identify superior oblique muscle with removal of all intervening tissues between it and the SR muscle for proper transposition.

The SR muscle was then secured close to its insertion using double armed 6/0 polyglactin sutures before it was disinserted from the globe. The undersurface of SR muscle was then inspected to remove any remaining attachment between it and the SO tendon or the frenulum. The SR muscle was laterally transposed and sutured to the sclera adjacent to the insertion of lateral rectus (LR) muscle along the spiral of Tillaux. Transposition was then dually augmented by muscle to muscle union suture coupled with a scleral pass adjacent to the border of the LR muscle and 10 mm posterior to its insertion using non absorbable 5/0 polyester suture. No adjustable suture was used in both MRc and SRT. Conjunctival closure was finally performed using 8/0 polyglactin sutures.

Postoperatively, angle of deviation in primary position, degree of AHP, limitation of ocular duction as well as postoperative complications were recorded at each follow up visit but only last visit’s findings were used for statistical analysis. Slit lamp examination was meticulously performed at each follow visit to detect any signs of anterior segment ischemia. Success was defined as within 10PD of horizontal orthotropia and within 4PD of vertical orthotropia with improvement of AHP. Significant induced vertical deviations of more than 4PD were considered surgical failure. Statistical software (SPSS for Windows V.17.0. Chicago: SPSS Inc.) was used for statistical analysis. Pre and postoperative numerical and categorical values were compared using *t* test and chi-square test respectively with a *P* value less than 0.05 considered statistically significant.

## Results

The review of medical records revealed a total of 21 patients, 10 patients with chronic sixth nerve palsy and 11 patients with Eso-DRS, who were treated by augmented SRT. In 18 patients, the SRT was combined with ipsilateral MRc (mean 4.7 mm, range; 4–5.5 mm), while in the remaining 3 cases; the SRT was done in isolation (supplementary table). In all cases, defective ocular abduction was unilateral.

Table 1 shows patients’ demographics as well as pre and postoperative clinical data. At the last postoperative visit, average ET in the primary position improved from 40.5PD and 28.6PD to 6.7PD ET and 2.5PD exotropia (*P* < .00001) while AHP improved from 31° and 28.1° to 4.5° and − 0.45° (*P* < .00001) in sixth nerve palsy and Eso-DRS groups respectively. Mean limitation of abduction improved from − 4.2 and − 3 to − 1.5 and − 1 units in sixth nerve palsy and Eso-DRS groups respectively (*P* < .00001) while in cases which underwent combined SRT and MRc, adduction declined by a mean of 0.7 (*P*= 0.021) and 0.4 unit (*P*=0.014) in sixth nerve palsy and Eso-DRS groups respectively. Stereopsis data could be obtained in 9 and 7 patients in the sixth nerve palsy and Eso-DRS groups, respectively. In both groups, no patient had a decrease in stereopsis with an overall increase in number of patients with fusion and stereopsis (Table 1).

**Table 1:** Demographics, pre and postoperative clinical data in both groups

Item	Six nerve palsy(n:10)	Esotropic DRS(n:11)
Age, mean, range, years	36.7 (8–64)	7.1 (4–12)
Sex		
male	7	2
female	3	9
Follow up, mean (range)	6.4 months (4–10)	6.7 months (3–11)
Combined MRc, mean (no. of cases)	4.8 mm (9 cases)	4.7 mm (10 cases)
Preoperative esotropia, mean (range)	40.5 PD (25–60)	28.6 PD (20–40)
Postoperative deviation, mean (range)	6.7 PD ET (6XT-15ET)	2.5XT (25XT-8ET)
Improved esotropia, mean (range)	33.8 PD (17–52)	31.1 PD (12–60)
Preoperative AHP, mean (range)	31° (20–45)	28.1° (15–50)
Postoperative AHP, mean (range)	4.5° (0–10)	−0.45° (5 to − 15)
Improved AHP, mean (range)	26.5° (20–35)	28.6 ° (15–50)
Preoperative abduction deficit, mean (range)	−4.2 (− 3.5 to − 5)	−3.09 (− 2 to − 4)
Postoperative abduction deficit, mean (range)	−1.5 (− 0.5 to − 3)	− 1.09 (0 to − 2)
Improved abduction deficit, mean (range)	2.6 (2–3)	2 (1–2.5)
Preoperative adduction, mean (range)	0.5 (0 to 1)	0
Postoperative adduction, mean (range)	−0.2 (− 1 to 0)	− 0.45 (− 1 to 0)
Induced adduction deficit, mean (rang)	0.7 (0–2)	0.4 (0–1)
Induced vertical deviation, number	4	3
Preoperative binocular vision, no., (%)		
Fusion	2/9 (22%)	4/7 (57%)
Stereopsis ≥3000 arcsc	1/9 (11%)	3/7 (42%)
Postoperative binocular vision, no. (%)		
Fusion	5/9 (55%)	6/7 (85%)
Stereopsis ≥3000 arcsc	3/9 (33%)	5/7 (71%)

*MRc* medial rectus recession, *AHP* abnormal head posture, *PD* prism diopter, *arcsc* arc second

**Fig. 1:**
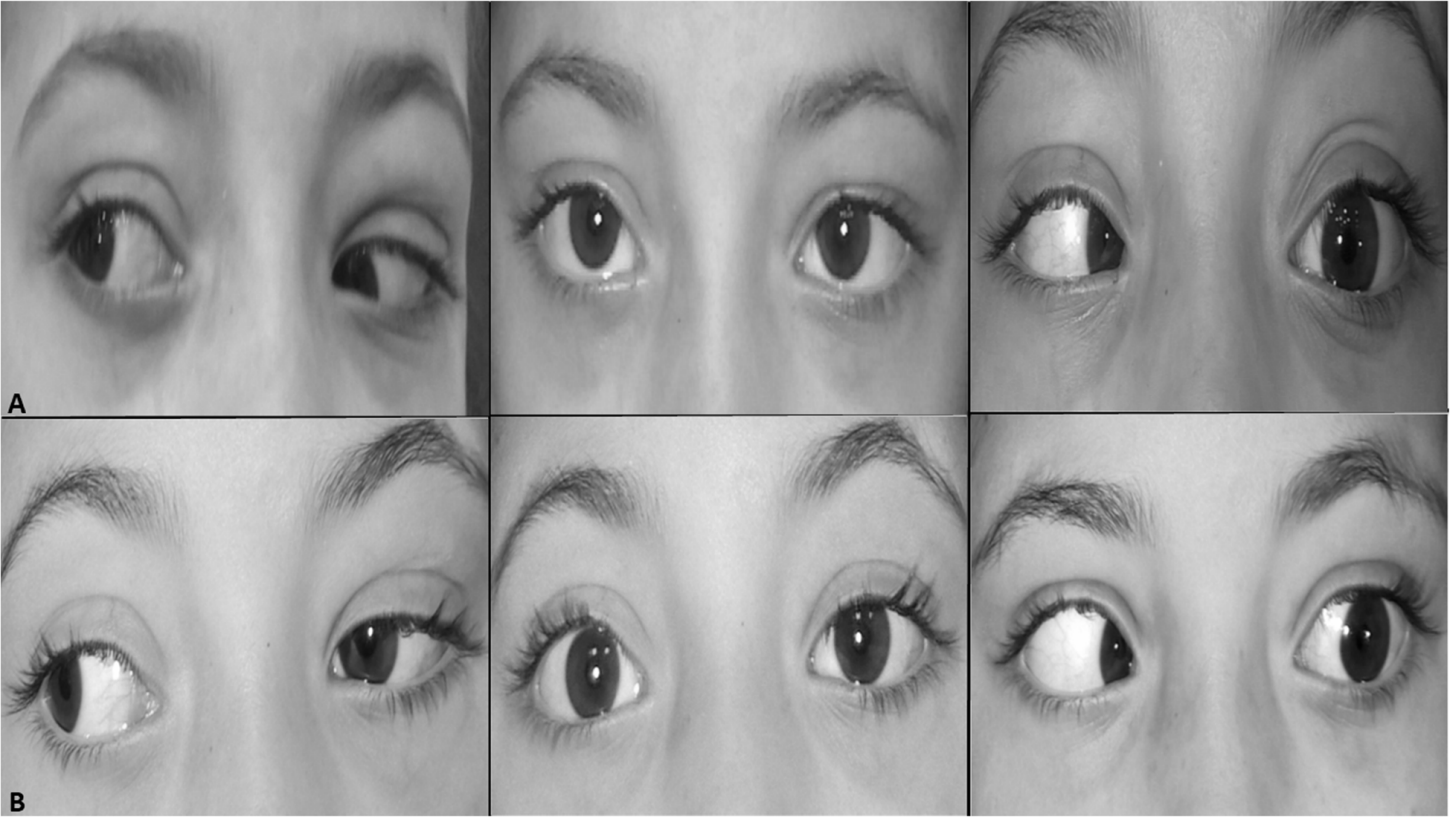
photos of horizontal gazes of a patient with left Esotropic Duane retraction syndrome (patient no.13; supplementary table) (**a**), preoperative photos with 25 PD esotropia and − 3.5 limited abduction in the left eye; **b**, postoperative photos showing 2PD exotropia, improvement of abduction to − 1 with induced limitation of adduction of − 1 in the left eye

**Fig. 2:**
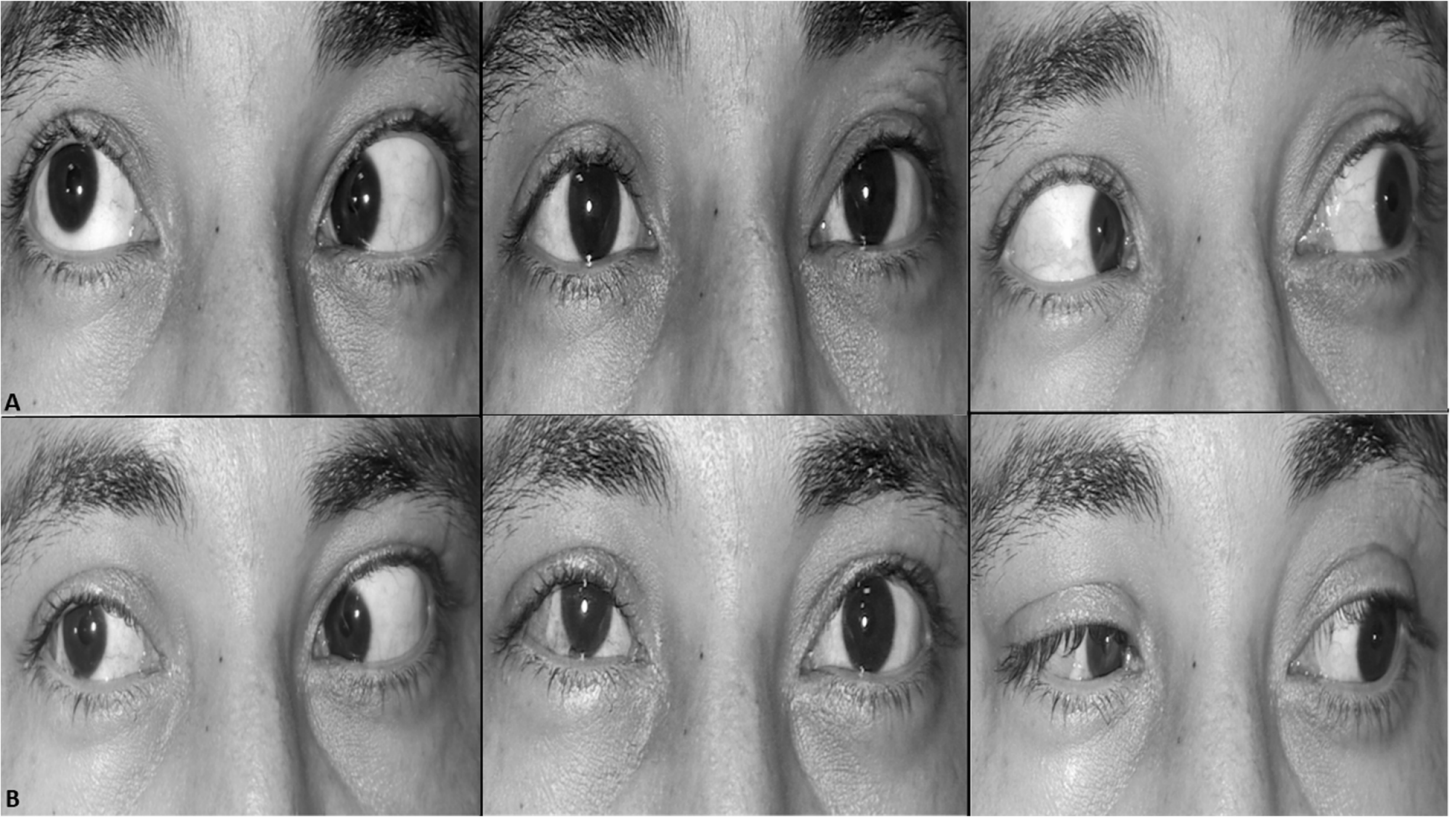
photos of horizontal gazes of a patient with right chronic 6th nerve palsy (patient no. 1; supplementary table) (**a**), preoperative photos with 35PD esotropia, 6PD hypertropia and − 3.5 limited abduction in the right eye; **b**, postoperative photos showing 12PD residual esotropia, 15PD induced hypertropia and improvement of abduction to − 0.5

**Fig. 3:**
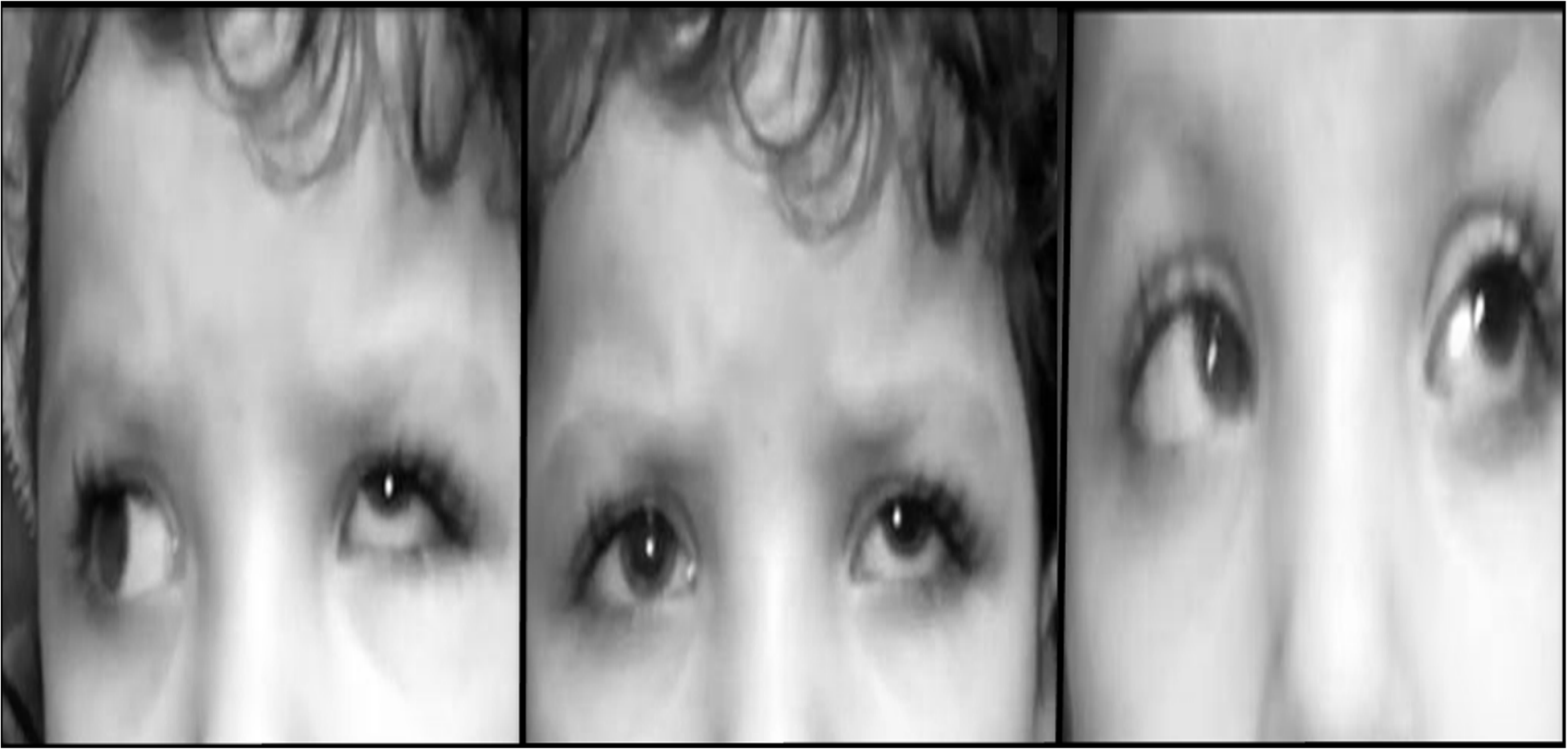
postoperative photographs of horizontal gazes of patient no. 15; supplementary table with left Esotropic Duane retraction syndrome complicated with large angle induced hypertropia of 25PD and consecutive exotropia of 25PD. The large angle induced hypertropia is constant across primary position, adduction and abduction

Success (within 10PD of horizontal orthotropia, within 4PD of vertical orthotropia with improvement of AHP) was achieved in 15 patients (6 patients in sixth nerve palsy group and 9 patients in ESO-DRS groups, Figs. 1 and 2). In all patients, single case of consecutive exotropia (supplementary table, patient no. 15) was recorded in Eso-DRS group while persistent ET was reported in 3 cases in the sixth nerve palsy group (supplementary table, patients no. 1, 7 and 10). Overall, 5 cases of significant induced hypertropia of more than 4PD were recorded in the two groups, 3 cases in sixth nerve palsy group and 2 cases in Eso-DRS group. In two cases (supplementary table, patient no. 1 and 15), the induced hypertropia was of high grade that patients were offered corrective interventions but both patients refused (Fig. 3). Two patients and 5 patients developed mild postoperative limitation of adduction in sixth nerve palsy and Eso-DRS groups respectively. No post-operative subjective torsional diplopia or signs of anterior segment ischemia were reported. Supplementary table shows detailed pre and postoperative data in all patients of both groups.

## Discussion

Various vertical rectus muscle transposition procedures have been proposed to overcome esotropia and anomalous head posture secondary to defective ocular abduction in chronic sixth nerve palsy and esotropic Duane retraction syndrome [4, 5, 11–18]. All those transposition procedures aimed at improvement of abduction with correction of esotropia and abnormal head position taking into consideration preservation of intact adduction and avoidance of anterior segment ischemia. In 2006, Johnston et al. were the first to propose transposition of superior rectus muscle to treat cases of esotropic DRS to decrease incidence of anterior segment ischemia, especially in cases where surgeries on multiple recti were planned [4]. The current study presents the results of augmented transposition of superior rectus muscle in treatment of chronic sixth nerve palsy and Eso-DRS. Overall, the procedure was found to be effective in controlling esotropia and AHP with significant correction of limited abduction.

In the current study, augmented SRT achieved an average correction of esotropia of 33.8 PD and 31.1 PD in sixth nerve palsy and esotropic DRS groups, respectively. Those figures closely match published results of SRT in previous studies [5, 11–18], in which, the average correction of ET varied between 19 to 51PD and 7 to 27PD in sixth nerve palsy and esotropic DRS, respectively (Table 2). Regarding limitation of abduction, the current study achieves 2.6 units of abduction improvement in sixth nerve palsy group and 2 units in Eso-DRS group. Comparatively, improvement in limitation of abduction in the current study is better than what has been achieved in previous reports which varied between 1.0 to 1.9 units of improved abduction in sixth nerve palsy and from 0.8 to 2 units of improved abduction in Eso-DRS. Table 2 compares the average changes in the main parameters of the current study with the previously published ones [5, 11–18]. Mild limitation of adduction was reported following combined SRT and MRc in the current study (Fig. 1) as well as in the previously published reports [5, 12–15, 18].

**Table 2:** Results of superior rectus muscle transposition in previous studies

Study, year	Total no. of patients	Patients’ population (number)	Procedure	ET, mean change	AHP, mean change	Abduction limitation, mean change	Adduction limitation, mean change	Induced vertical deviation, number
**Mehendale** [5]**, 2012**	17	Eso-DRS (10)	^a^Augmented SRT+MRc	27	21	2	0.6	0
		Sixth nerve palsy (7)	^a^Augmented SRT+MRc	36.7	31	1.8	0.6	1 HOT 1 N/A
**Yang** [13]**, 2104**	19	Eso-DRS	^a^Augmented SRT±MRc	26	20	1.5	0.6	2 HOT
**Velez** [17]**, 2014**	11	Eso-DRS (4)	Augmented SRT±MRc	26	9.3 ^b^	1.4 ^b^	N/A	1 HT 3 HOT
		Sixth nerve palsy (7)	Augmented SRT±MRc	38.6	9.3 ^b^	1.4 ^b^	N/A	5 HT
**Tibrewal** [14]**, 2105**	8	Eso-DRS	Augmented SRT+MRc	17	12	1.2	1	0
**Preeti Patil-Chhablani** [15]**, 2016**	13	Sixth nerve palsy	Augmented SRT+MRc	45.5	14.4	1.9	0.1	1 HOT
**Yeon-Hee Lee** [12]**, 2107**	8	Sixth nerve palsy	Augmented SRT+MRc	36.4	N/A	1.6	0.7	0
**Yan-Liu** [11]**, 2108**	13	Sixth nerve palsy	Augmented SRT±MRc	51.4	N/A	1.5	N/A	2 HT
**Akbari** [16]**, 2018**	22	Eso-DRS (11)	Augmented SRT	7.8	5.9	0.8	N/A	3 HOT
		Sixth nerve palsy (11)	Augmented SRT	19.2	14.5	1.0	N/A	4 HOT
**Agarwal** [18]**, 2018**	19	Eso-DRS (9)	SRT±MRc	23.9	16	2.0	0.6	0
		Sixth nerve paly (10)	^a^Augmented SRT+MRc	45.4	17	1.8	0	0
**Present study, 2020**	21	Eso-DRS (11)	Augmented SRT±MRc	31.3	28.6	2	0.4	2 HT
		Sixth nerve palsy (10)	Augmented SRT±MRc	29.8	22.5	2.5	0.7	3 HT

*SRT* superior rectus transposition, ^a^ augmentation was performed in some patients only, as per author preference, *ET* esotropia, *AHP* abnormal head posture, *HOT* hypotropia, *HT* hypertropia, *N/A* not available, ^b^ average improvement of the item in the whole study population as specific values in individual groups were not clarified

Induced vertical deviations were documented following SRT in some previous studies and in the current study as well [5, 11–13, 15–17]. In some previous reports [5, 13, 16], induced vertical deviation was in the form of hypotropia which was assumed to be secondary to diminished SR vertical force vector which would prone the eye to postoperative hypotropic drifts. However, the incidence of induced hypotropia was found to be less frequent than expected, and the proposed explanation for that was a counter increase in the SR strength induced by its anterior advancement during transposition to follow the spiral of Tillaux [13]. In some other reports, SRT- induced vertical deviation was in the form of hypertropia [11, 17, 19]. In Velez et al. study which included 11 patients with sixth nerve palsy and esotropic DRS [17], induced hypertropia was recorded in 6 patients and in half of them, the magnitude of hypertropia was quite large (7PD in two cases and 10PD in one case). In Yan Liu’s study [11], 10PD induced hypertropia developed in two cases. Interestingly, hypertropia completely disappeared in one case after subsequent transposition of the ipsilateral inferior rectus performed 2 months after the primary procedure. Merino at al [18] published a case of symptomatic 12PD induced hypertropia following augmented SRT combined with MRc in a patient with unilateral Eso-DRS. Similarly, hypertropia improved to 4PD after 2 mm recession of the transposed SR combined with removal of posterior augmentation suture. In the current study, severe overcorrection of horizontal deviation was reported in one case only and, in accordance with previous reports [5, 17], this case was a 4 years old child with esotropic DRS (Fig. 3 and supplementary table).

Improvement of SRT-induced hypertropia following subsequent transposition of ipsilateral inferior rectus muscle or revision of the transposition procedure in previous reports should raise questions about the exact mechanism of induced vertical deviations following SRT [11, 18]. We believe that such induced vertical deviation which developed after this form of “single” vertical rectus muscle transposition was probably secondary to the unbalanced vertical force vector which originated following isolated transposition of one of the vertical recti. In addition, one of the main advantages of SRT is to spare anterior ciliary arteries thus decreasing the theoretical incidence of anterior segment ischemia, especially when multiple rectus muscle surgery was required [6]. This notion is well understood if SRT is to be compared with full tendon vertical recti transposition. However, this advantage would be less appealing when SRT is to be compared with partial thickness VRT, as both procedures would sacrifice only two anterior ciliary arteries [20] while partial VRT seems more anatomically balanced. In a previous report [9], augmented partial VRT in chronic sixth nerve palsy has resulted in 3 cases of mild induced hypertropia ranged 2 to 4PD only.

## Conclusion

In conclusion, augmented SRT with or without MRc has a positive impact on esotropia, AHP and limited abduction secondary to chronic sixth nerve palsy and Eso-DRS. However, high rates and degrees of induced hypertropia reported in the current study highlight the unbalanced nature of SRT. We believe that SRT should be judged in future trials against its actual contender in the market of vertical rectus muscle transposition, partial VRT, to determine which procedure will give the best results with lowest possible incidence of induced vertical deviations.

## Supplementary Information

**Additional file 1: Supplementary Table 1** Demographics and Clinical characteristics of patients in both groups.

## Data Availability

The datasets used and/or analysed during the current study are available from the corresponding author on reasonable request.

## Abbreviations

AHP: Abnormal Head Posture
Eso-DRS: Esotropic Duane retraction syndrome
ET: Esotropia
LR: Latreal Rectus
MR: Medial Rectus
MRc: Medial Rectus recession
PD: Prism Diopters
SO: Superior Oblique muscle
SRT: Superior Rectus Transposition
VRT: Vertical Rectus Transposition
