# Supplementary material for: Augmented superior rectus muscle transposition in management of defective ocular abduction
Source: BMC Ophthalmol. 2021 Jan 20;21:50. doi: 10.1186/s12886-020-01779-1 (PMC7818568; doi:10.1186/s12886-020-01779-1)
Supplement: Supplementary file 1 — Additional file 1: Supplementary Table 1 Demographics and Clinical characteristics of patients in both groups. [file 12886_2020_1779_MOESM1_ESM.docx]

| **patient** | **Age** | **Sex** | **side** | **FU** | **procedure** | **Deviation**  **preop postop** | | **AHP**  **preop postop** | | **Abduction**  **preop postop** | | **Adduction**  **preop postop** | |
| --- | --- | --- | --- | --- | --- | --- | --- | --- | --- | --- | --- | --- | --- |
| **Sixth nerve palsy** | | | | | | | | | | | | | |
| 1 | 35 | M | OD | 4 | SRT | 35ET  6HT | 12ET 15HT | 25° | 5° | -3.5 | -0.5 | 0 | 0 |
| 2 | 23 | M | OD | 8 | SRT+MRc 5mm | 35ET | 4ET | 35° | 10° | -4 | -2 | 1 | -1 |
| 3 | 8 | F | OS | 4 | SRT | 25ET | 8ET | 25° | 5° | -3.5 | -1 | 0 | 0 |
| 4 | 61 | F | OS | 6 | SRT+MRc 5mm | 45ET | 8ET 4HT | 30° | 5° | -5 | -2 | 1 | 0 |
| 5 | 16 | M | OS | 10 | SRT+MRc 4mm | 30ET | 6ET | 20° | 0 | -4 | -2 | 0 | 0 |
| 6 | 45 | M | OD | 5 | SRT+MRc 5mm | 50ET | 2XT | 35° | 0 | -4 | -1 | 1 | 0 |
| 7 | 54 | M | OS | 9 | SRT+MRc 4.5mm | 40ET | 12ET | 30° | 0 | -4 | -1 | 0 | 0 |
| 8 | 32 | M | OD | 7 | SRT+MRc 5mm | 50ET | 10ET 8HT | 35° | 5° | -5 | -2 | 1 | 0 |
| 9 | 29 | F | OD | 5 | SRT+MRc 4.5mm | 35ET | 6XT | 30° | 10° | -4 | -1 | 0 | 0 |
| 10 | 64 | M | OS | 6 | SRT+MRc 5.5mm | 60ET | 15ET 6HT | 45° | 5° | -5 | -3 | 1 | -1 |
| **Esotropic Duane retraction syndrome** | | | | | | | | | | | | | |
| 11 | 4 | F | OS | 5 | SRT+MRc 4mm | 20ET | 2ET | 15° | 0 | -2 | 0 | 0 | -1 |
| 12 | 6 | F | OD | 6 | SRT+MRc 5mm | 30ET | 2ET 4HT | 25° | 0 | -3 | -1 | 0 | 0 |
| 13 | 6 | F | OS | 8 | SRT+MRc 4.5mm | 25ET | 2XT | 50° | 0 | -3.5 | -1 | 0 | -1 |
| 14 | 5 | F | OS | 5 | SRT+MRc 5mm | 40ET | 10XT | 35° | -5° | -4 | -2 | 0 | -1 |
| 15 | 4 | F | OS | 4 | SRT+MRc 5mm | 35ET | 25XT 25HT | 30° | -15° | -4 | -2 | 0 | -1 |
| 16 | 11 | F | OS | 8 | SRT+MRc 5mm | 30ET | 6XT | 25° | 0 | -3 | -1 | 0 | -1 |
| 17 | 7 | F | OS | 11 | SRT+MRc 5mm | 35ET | 6ET 5HT | 30° | 5° | -3.5 | -1 | 0 | 0 |
| 18 | 9 | F | OD | 3 | SRT | 20ET | 8ET | 25° | 5° | -3 | -1 | 0 | 0 |
| 19 | 6 | F | OS | 7 | SRT+MRc 4mm | 25ET | 1 ET | 15° | 0 | -2 | 0 | 0 | 0 |
| 20 | 9 | F | OD | 10 | SRT+MRc 5mm | 30ET | 4XT | 40° | 5° | -4 | -2 | 0 | 0 |
| 21 | 12 | F | OS | 7 | SRT+MRc 4.5mm | 25ET | Ortho | 20° | 0 | -2 | -1 | 0 | 0 |

**Supplementary table; Demographics and Clinical characteristics of patients in both groups**

SRT; superior rectus transposition, MRc; medial rectus recession, ET; esotropia, XT; exotropia, HT; hypertropia, AHP; abnormal head posture, DS; downshoot, US; upshoot
